# Knowledge about human papillomavirus and the HPV vaccine – a survey of the general population

**DOI:** 10.1186/1750-9378-4-S1-S10

**Published:** 2009-02-10

**Authors:** Camille C Ragin, Robert P Edwards, Jade Jones, Natalie E Thurman, Kourtney L Hagan, Erin A Jones, Cierra M Moss, Ar'Lena C Smith, Aletha Akers, Susanne M Gollin, Dwight E Heron, Cecile Andraos-Selim, Cornelius Bondzi, Linda Robertson, Emanuela Taioli

**Affiliations:** 1 Department of Epidemiology, University of Pittsburgh Graduate School of Public Health, Pittsburgh, PA, USA; 2 Division of Cancer Prevention and Population Science, University of Pittsburgh Cancer Institute, Pittsburgh, PA, USA; 3 Division of Gynecologic Oncology, University of Pittsburgh Cancer Institute, Pittsburgh, PA, USA; 4 Department of Biological Sciences, Hampton University, Hampton, VA, USA; 5 Division of Gynecologic Specialties, Department of Obstetrics, Gynecology & Reproductive Sciences, University of Pittsburgh, Pittsburgh, PA, USA; 6 Department of Human Genetics, University of Pittsburgh Graduate School of Public Health, Pittsburgh, PA, USA; 7 Department of Radiation Oncology, University of Pittsburgh Cancer Institute, Pittsburgh, PA, USA; 8 Department of Community and Behavioural Health Science, University of Pittsburgh Graduate School of Public Health, Pittsburgh, PA, USA; 9 Department of Cancer Control, University of Pittsburgh Cancer Institute, Pittsburgh, PA, USA; 10 Department of Epidemiology and Biostatistics, Downstate School of Public Health, State University of New York, USA

## Abstract

**Background:**

The United States (US) Food & Drug Administration (FDA) recently approved a human papillomavirus (HPV) vaccine with the purpose of reducing the risk of cervical cancers caused by HPV 16 and HPV 18. It is important that the general population be educated about HPV and the HPV vaccine in order to make the appropriate decision whether or not to vaccinate against this virus. Participants from the adult US general population of Pittsburgh, Pennsylvania, USA and Hampton, Virginia, USA (18+ years old) were surveyed to determine their knowledge about HPV and the HPV vaccine, and to evaluate their perception of the vaccine efficacy and safety.

**Results:**

We report herein preliminary data for 202 participants. Fifty-five percent (55%) of the study population was White, 45% Black, and 1% was from other ethnic groups or did not disclose their ethnicity. A large proportion of participants had heard of the human papillomavirus (overall population: 93.6%; Pittsburgh: 95%; Hampton: 90%). Participants of African descent were slightly less aware of HPV than Whites (Black 89% vs. Whites 97%, p > 0.1). Although the majority of participants knew that HPV caused cervical cancer (84%), Whites were more informed than Black participants (91% vs. 73%, p = 0.044). Eighty-seven percent (87%) of participants had heard of the HPV vaccine (Pittsburgh: 92% and Hampton: 74%, p = 0.029); a higher proportion of Whites were aware of the vaccine when compared with Blacks (93% vs. 76%, p = 0.031). However, only 18% of the population knew that the current FDA-approved vaccine protected against genital warts and most cervical cancer (20% of Blacks and 16% of Whites, p > 0.1).

**Conclusion:**

These data suggest that although the general population might be aware of HPV and the HPV vaccine, knowledge of the benefits of the HPV vaccination may not be apparent. Knowledge of HPV and the HPV vaccine could result in a likely choice of HPV vaccination and would subsequently reduce the incidence of cervical cancer.

## Background

Human papillomavirus (HPV) is a family of viruses that are etiologically linked to number of disease conditions: cancers as well as benign conditions such as warts and condylomas. Although there are treatments for the clinical manifestations of HPV infection, there is no cure for the infection. There are at least 15 high-risk HPV types that cause cervical cancer and most anogenital cancers; HPV types 16 and 18 are the most common. In addition, HPV16 is thought to be linked to the development of a subset of head and neck cancers [1,2].

The United States (US) Food & Drug Administration (FDA) recently approved an HPV vaccine with the purpose of reducing the risk of cervical cancers caused by HPV 16 and HPV 18 [3]. The Centers for Disease Control and Prevention (CDC) Advisory Committee on Immunization Practices (ACIP) recommended routine HPV vaccination for 11–12 year olds and for girls as young as 9 years old. The committee also recommended the vaccination of 13–26 year old females who have not been previously vaccinated or have not completed the full vaccination series [4].

The HPV vaccine prevents infections from HPV types 6, 11, 16 and 18. HPV types 6 and 11 are primarily responsible for the development of genital warts, while HPV types 16 and 18 are attributed to the development of approximately 70% of all cervical cancers [5,6]. The CDC has made the HPV vaccine available through the Federal Vaccines for Children (VFC) program in all 50 states [7]. Currently there are no federal laws that require children or adolescents to be vaccinated. The only state that has mandated HPV vaccination of girls for school attendance is Virginia. However, this state is currently considering a bill that will delay that requirement [8]. Nevertheless, for many states, HPV vaccination is voluntary. Therefore, it is important that the general population be educated about HPV and the HPV vaccine so that an informed decision can be made as to whether or not to vaccinate against this virus.

We have developed a survey that assesses the general population's knowledge and perception of HPV as well as the HPV vaccination. This report summarizes data collected from a sample of the general population residing in Pittsburgh, Pennsylvania, USA and Hampton, Virginia, USA. We have compared the knowledge and attitudes between the populations from these states.

## Methods

### Study population

The survey was administered to participants from the general population of Pittsburgh, Pennsylvania and Hampton, Virginia, 18 years of age and older. Insitutional Review board approval was obtained from both the University of Pittsburgh and Hampton University. The surveys were distributed face-to-face or by mail. The Pittsburgh population consisted of participants who were enrolled in a population-based registry of people from Pittsburgh and surrounding communities. These participants were recurited through flyers posted at churches, community centers, outpatient clinics, health fairs, school events, libraries, the local newspapers, and the University of Pittsburgh Medical Center newsletters. A letter of invitation to participate was mailed along with the questionnaire and interested participants completed and returned the survey by mail. A total of 209 (192 Whites, 14 Blacks and 2 from other ethnic groups) surveys were mailed and 103 participants (49.3%) responded (98 Whites, 2 Blacks, one from another ethnic group and two who did not disclose their ethnicity). Additional participants (N = 23, 19 Blacks and 4 Whites) were recruited face-to-face, from an ongoing study on participants who were at one time seen as patients at the Magee Womens Hospital. The response rate for the face-to-face recruitment was 23/60 (38%). The Hampton population consisted of participants from Hampton and surrounding communities, such as the local plasma donation center, churches, the local police station, and also students and staff members from the Hampton University campus (68 Blacks, 7 Whites and one from another ethnic group). These participants were recruited face-to-face. All of the surveys for both study populations were completed anonymously.

### Questionnaire

The questionnaire development was guided by our research hypotheses and a review of the literature, and consists of questions that define the participant's age, gender, ethnicity, birthplace, marital status, parental status, number of children, level of education, religion, health insurance coverage and yearly household income. Participants were also asked to answer questions that would evaluate their level of knowledge of HPV and the HPV vaccine. Questions related to the participant's perception of the safety, efficacy and impact of HPV vaccination were also included in the survey. A preliminary content assesment of the survey was performed prior to the initiation of this study.

### Statistical analysis

All statistical analyses were performed using STATA SE (version 10) software (StataCorp LP, College Station, TX, USA). A two-sample proportions test of significance was performed when comparing demographic variables and the survey answers between stratified groups. Comparisons of continuous variables were performed using the two-sample t-test. A p-value < 0.05 was considered statistically significant. Adjusted prevalence rates for each survey answer were calculated for all participants and stratified by ethnic groups and population source (Pittsburgh and Hampton) after adjusting for potential confounding variables, such as age, marital status, education, income, recruitment location, and parental status. Comparisons of the number of correct answers for HPV knowledge and HPV vaccine knowledge questions stratified by race and recruitment location were calculated using two-way ANOVA models after adjusting for age, marital status, education, income, recruitment location and parental status. Box plots were generated using the adjuste mean vaules for each stratifed model.

## Results

### Description of the study populations

A total of 202 participants completed the survey (62% from Pittsburgh and 38% from Hampton – Table 1). The Hampton population consisted of younger individuals compared with the Pittsburgh population (Hampton: 18–35 = 87%, 36–55 = 11%, 56+ = 3% vs. Pittsburgh: 18–35 = 20%, 36–55 = 45%, 56+ = 35%). Overall, 54.5% of the study population was White, 44.5% Black, and 1% was from other ethnic groups or did not disclose their ethnicity. While the majority of White participants were born in the United States (98%), 2% were immigrants born in Sub-Saharan Africa (Angola) or Australia. Among the Black population, 5% were immigrants born in Germany, Africa (Ghana), Jamaica and the U.S. Virgin Islands. All others were African-American (95%). Twenty-eight percent of participants were male and 72% were female. The majority of participants had some level of post-secondary education (92%), had access to healthcare (94% had health insurance coverage), and 51% had a yearly household income greater than US$55,000. The majority of Black participants were recruited from Hampton, VA (76% vs. 24%, p = 0.0001) while the majority of White participants were from Pittsburgh, PA (94% vs. 6%, p < 0.0001). We observed no differences in gender, education level, access to health care, income, sexual activity or religion between Black and White participants. However, 74% of the Black participants were between the ages of 18 and 35 years and were statistically significantly younger than the White participants (22%, p < 0.0001). The majority of Blacks were single compared with Whites (75% vs. 25%, p < 0.0001), while the majority of Whites were married compared with Blacks (58% vs. 15%, p = 0.006). In addition, the majority of Whites (64%) were parents compared with 33% of Black participants (p = 0.005). The average number of children for each parent was significantly higher for Blacks compared with Whites (3 vs. 2 children per parent, p = 0.0001).

**Table 1:** Description of the studied populations according to race

**All respondents N (%)**		**Blacks N (%)**	**Whites N (%)**	**Other N (%)**	p-value*
	202 (100)	89 (44.5)	109 (54.5)	2 (1.0)	
**Location**					
Hampton, VA	76 (37.6)	68 (76.4)	7 (6.4)	1 (50.0)	0.0001
Pittsburgh, PA	126 (62.4)	21 (23.6)	102 (93.6)	1 (50.0)	<0.0001
**Age**					
18–35	90 (45)	65 (73.9)	24 (22.4)	1 (50.0)	<0.0001
36–55	63 (32)	20 (22.7)	42 (39.3)	-	
56+	45 (23)	3 (3.4)	41 (38.3)	1 (50.0)	
**Gender**					
Female	143 (72.2)	61 (69.3)	80 (74.8)	1 (50.0)	
Male	55 (27.8)	27 (30.7)	27 (25.2)	1 (50.0)	
**Parent**					
Yes	99 (49.0)	28 (32.6)	70 (64.2)	1 (50.0)	0.005
Children (average no, range)	2, 1–5	3, 1–5	2, 1–4	3	0.0001
**Education level**					
High school or less	16 (8)	9 (10.2)	7 (6.4)	-	
Post-secondary education	184 (92)	79 (89.8)	102 (93.6)	2 (100.0)	
**Annual household income**					
Less than $35,000	57 (31.5)	32 (39.0)	23 (24.0)	1 (50.0)	
$25,000–$55,000	32 (17.7)	15 (18.3)	17 (17.7)	-	
Greater than $55,000	92 (50.8)	35 (42.7)	56 (58.3)	1 (50.0)	
**Health Insurance**					
No	12 (6.0)	8 (9.0)	3 (2.8)	1 (50.0)	
Yes	189 (94.0)	81 (91.0)	106 (97.2)	1 (50.0)	
**Religion**					
Christian	150 (77.3)	67 (79.8)	82 (75.9)	1 (50.0)	
Other	28 (14.4)	15 (17.9)	12 (11.1)	1 (50.0)	
None	16 (8.3)	2 (2.3)	14 (13.0)	-	
**Marital status**					
Single	96 (47.8)	67 (75.3)	27 (24.8)	1 (50.0)	<0.0001
Married	77 (38.3)	13 (14.6)	63 (57.8)	1 (50.0)	0.006
Other	28 (13.9)	9 (10.1)	19 (17.4)	-	
**Sexually active**					
No	66 (33.3)	26 (30.2)	37 (33.9)		
Yes	131 (66.2)	60 (69.8)	71 (65.1)		

*p-value shown only when there is a statistically significant difference between Blacks vs. Whites; Race was not defined for 2 participants

### HPV knowledge

All study participants were asked to disclose whether or not they had heard of the human papillomavirus and then asked to complete 10 additional questions that served to evaluate the level of HPV knowledge. The proportion of participants that provided answers to these questions were calculated after adjusting for potential confounders and summarized in Table 2. Overall, a large proportion of participants had heard of the human papillomavirus (overall study population: 93.6%; Pittsburgh: 95%, and Hampton: 90%). Blacks were slightly less aware of HPV than Whites, however the proportions were not statistically significantly different from each other (Blacks 89% vs. Whites 97% – Table 2). For the 10 questions that assessed HPV knowledge, in general, Blacks were less knowledgeable than Whites (Table 2).

**Table 2:** Assessment of HPV knowledge: adjusted proportions of the correct answers by race

	Blacks* (95% CI)	Whites* (95% CI)	**p-value**
Have you heard of the Human Papillomavirus (HPV)? *Answer = Yes*	89.3% (75.5%–95.8%)	96.9% (90.6%–99.0%)	
HPV is not sexually transmitted *Answer = False*	63.9% (47.7%–77.4%)	81.0% (69.6%–88.8%)	
HPV infection is relatively uncommon *Answer = False*	63.8% (47.6%–77.4%)	87.7% (78.4%–93.3%)	0.013
HPV causes cervical cancer *Answer = True*	73.2% (57.2%–84.8%)	90.7% (81.6%–95.5%)	0.044
Who can become infected with HPV? *Answer = Both women and men*	54.4% (38.7%–69.2%)	65.1% (51.5%–76.6%)	
Both men and women can have cervical cancer *Answer = False*	83.6% (67.9%–92.5%)	95.8% (87.4%–98.7%)	
The incidence of HPV in women is highest among women in their 20's and 30's *Answer = True*	69.2% (53.9%–81.2%)	65.9% (52.2%–77.4%)	
Most people with genital HPV infections are symptomatic *Answer = False*	41.5% (26.5%–58.3%)	73.6% (60.5%–83.5%)	0.014
HPV causes genital warts *Answer = True*	37.3% (24.1%–52.7%)	51.7% (38.6%–64.5%)	
Genital warts are caused by the same HPV types that cause cervical cancer *Answer = False*	19.5% (10.3%–33.8%)	16.5% (8.7%–28.9%)	
There is a cure for HPV infection *Answer = False*	47.6% (31.2%–64.6%)	68.4% (54.3%–79.8%)	

*Proportions adjusted for location, age, education, income, parental status and marital status; p-value shown only when there is a statistically significant difference between Blacks and Whites.

Overall, 74% of the study population knew that HPV was sexually transmitted. Although Black participants were less aware of this information (64%), compared with 81% of White participants, this difference was only marginally statistically significant (p = 0.104). Statistical significant differences were observed between Blacks and Whites for three specific questions. Only 64% of Black participants knew that HPV infection was relatively common compared with 88% of White participants (p = 0.013). Similarly, 48% of Black participants knew that there was no cure for HPV infection compared with 68% of Whites (p = 0.044). Furthermore, although a large proportion of participants knew that HPV caused cervical cancer (84%), Whites were more informed than Blacks (91% vs. 73%, p = 0.044).

For the majority of questions (8/10), the proportion of Black participants that answered correctly was less 70% (range: 19.5%–69.2). In contrast, for 5/10 questions, the proportion of White participants that knew the correct answers was less than 70% (range: 16.5%–68.4%). There were four questions that both Black and White participants answered poorly. Only 37% of Blacks and 52% of White participants knew that HPV caused genital warts; 20% of Blacks and 17% of Whites knew that genital warts were not caused by the same HPV type that caused cervical cancer. Furthermore, when asked: "Who can become infected with HPV?" only 54% of African-Americans and 65% of Whites knew that both men and women could be infected. Most importantly, only 69% of African-Americans and 66% of Whites were aware that the incidence of HPV in women is highest among women in their 20 s and 30 s.

**Figure 1:**
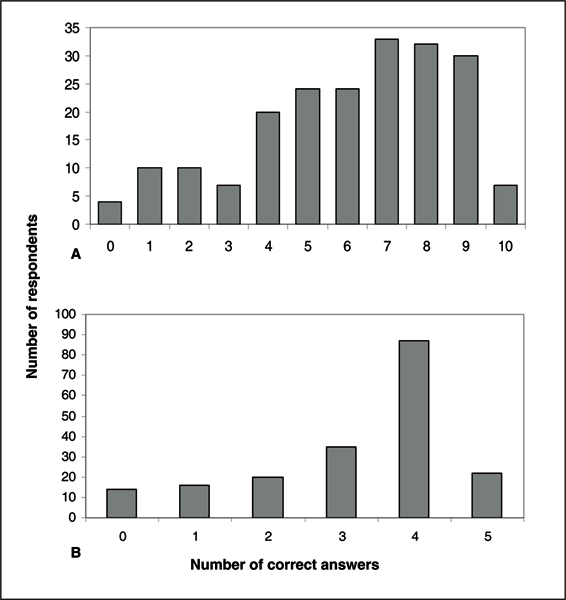
Cumulative distribution of the number of respondents who provided correct answers to the questions on the level of knowledge for (A) HPV and (B) the HPV vaccine.

**Figure 2:**
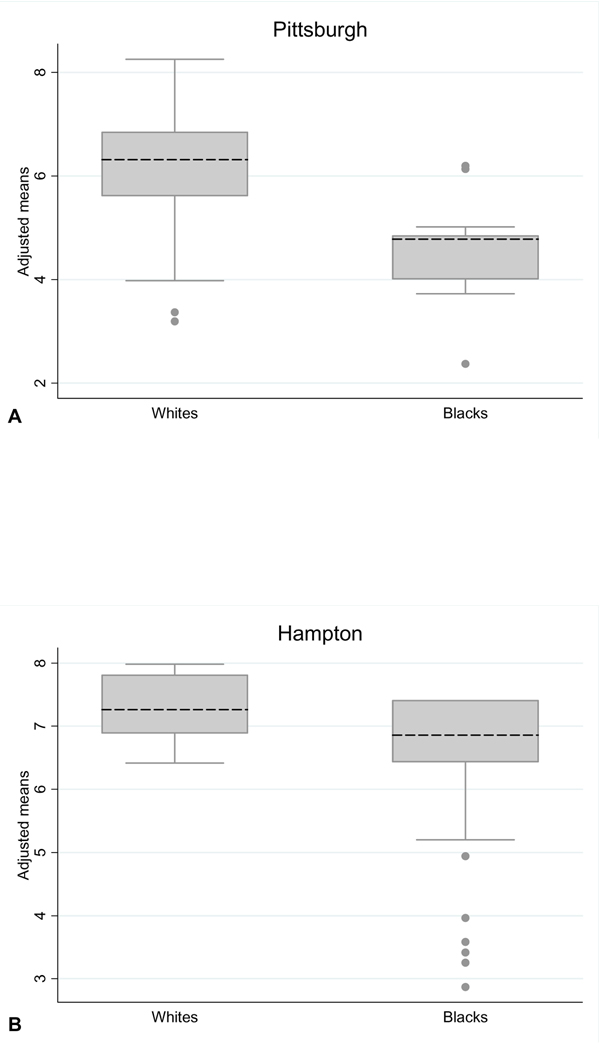
Distribution of the predicted number of correct answers to the 10 questions which assessed HPV knowledge, stratified by ethnicity and recruitment location and adjusted for age, education, income, parental status and marital status. The adjusted mean for each stratum is denoted by dashed lines.

Figure 1A shows cumulatively the number of respondents who provided correct answers to the 10 HPV knowledge-related questions. Among the participants who provided correct answers, the majority were successful in answering at least 7 of the 10 questions. Figure 2 shows the distribution of correct answers stratified by race and recruitment site. After adjusting for age, education, income, parental status and marital status, the overall mean number of correct answers for the Hampton participants was higher compared with participants from Pittsburgh (adjusted mean: 6.7 vs. 6.0). However, this difference was not statistically significant (p = 0.890). Within each recruitment site (Figure 2A,B), there was no difference between Blacks and Whites from Hampton (adjusted mean: 6.6 vs. 7.3, p = 0.707), however, in Pittsburgh, the mean number of correct answers for Blacks was lower than Whites (adjusted mean: 4.5 vs. 6.3, p = 0.032).

### Knowledge and perception of the HPV vaccine

Five questions were posed for the knowledge assessment of the HPV vaccine, summarized in Table 3. Similarly to the HPV knowledge assessment, proportions of participants that provided the correct answers were calculated after adjusting for potential confounders. For four of the five questions, a lower proportion of Blacks answered the HPV vaccine knowledge questions correctly compared with Whites. When participants were asked whether they had heard of the HPV vaccine, 87% answered affirmatively (Pittsburgh: 92% and Hampton: 74%, p = 0.029) and a higher proportion of Whites were aware of the vaccine compared with Blacks (93% vs. 76%, p = 0.031). However, despite this heightened awareness, only 18% of the overall study population knew that the current FDA-approved vaccine protected against genital warts and most cervical cancer (20% of Blacks and 16% of Whites – Table 3).

**Table 3:** Knowledge assessment of the HPV vaccine: adjusted proportions of the correct answers by race

	Blacks* (95% CI)	Whites* (95% CI)	**p-value**
Have you heard about the HPV vaccine? *Answer = Yes*	76.4% (60.1%–87.4%)	93.3% (84.9%–97.2%)	0.031
The HPV vaccine is approved for individuals who have never been infected with HPV *Answer = True*	48.8% (33.8%–64.0%)	81.2% (69.9%–88.9)	0.004
Who is eligible for the HPV vaccine? *Answer = Females*	63.7% (47.8%–77.1%)	81.3% (69.2%–89.3%)	
For which age group is the HPV vaccine recommended? *Answer = 9–26*	63.2% (46.6%–77.2%)	89.5% (78.5%–95.2%)	0.012
The current FDA approved HPV vaccine protects against genital warts and most cervical cancer *Answer = True*	20.0% (10.0%–36.1%)	16.1% (8.1%–29.3%)	
Once vaccinated women no longer have to be screened (annual pap smears) for cervical cancer *Answer = False*	78.9% (63.8%–88.8%)	91.7% (82.7%–96.3%)	

*Proportions adjusted for location, age, education, income, parental status and marital status; p-value shown only when there is a statistically significant difference between Blacks and Whites.

Although marginally statistically significant, a lower proportion of Blacks were aware that only females were eligible for the HPV vaccine (64%) at this time compared with 81% of White participants (p = 0.106). In contrast, statistically significant differences were observed for other questions. Forty-nine percent of Blacks compared with 81% of Whites knew that the HPV vaccine was approved for participants who have never been infected with HPV (p = 0.004). Furthermore, 63% of Blacks and 90% of Whites knew that 9–26 years was the correct age group for which the HPV vaccine was recommended (p = 0.012).

**Figure 3:**
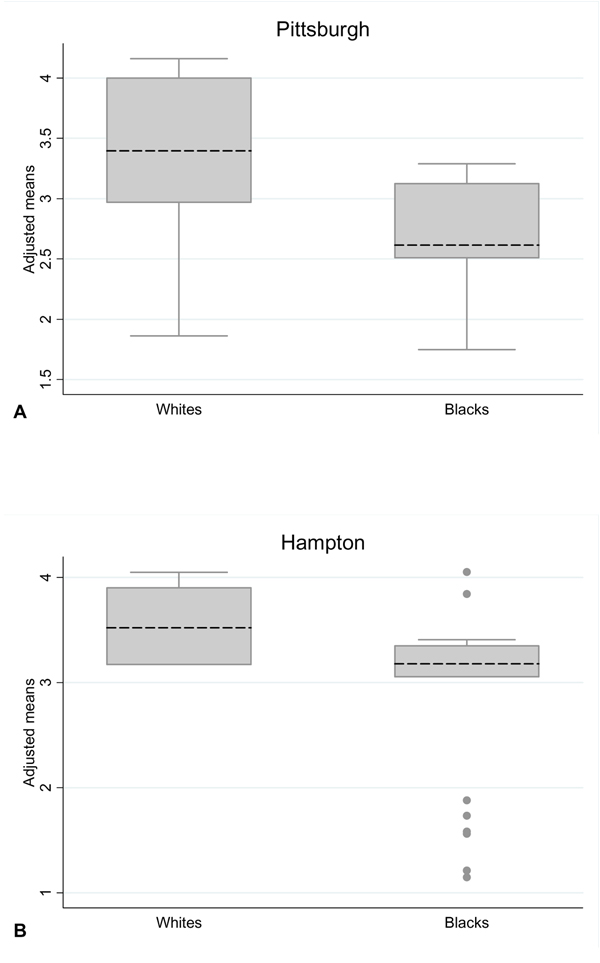
Distribution of the predicted number of number of correct answers to the five questions which assessed knowledge of the HPV vaccine, stratified by ethnicity and recruitment location and adjusted for age, education, income, parental status and marital status. The adjusted mean for the number of correct answers in each stratum is denoted by dashed lines.

Among the participants that provided correct answers to the HPV vaccine knowledge questions, the majority of participants were able to answer four out of the five questions correctly (Figure 1B). Overall, the mean number of correct answers were the same for Pittsburgh and Hampton participants (adjusted mean: 3.2 vs. 3.1, p = 0.883). Regardless of recruitment site, White participants appeared to be more knowledgeable than Black participants, since the mean number of correct answers was higher in White participants recruited from both Pittsburgh (3.4 vs. 2.7, p = 0.001) and Hampton (3.6 vs. 3.0, p = 0.887) (Figure 3A,B).

**Figure 4:**
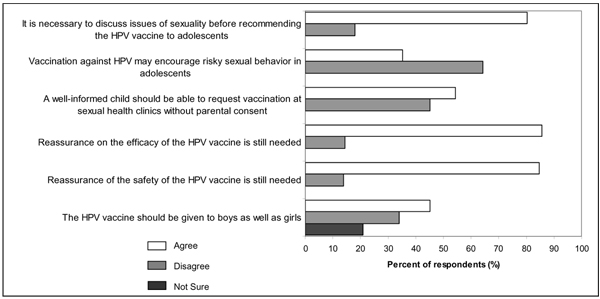
Participants' responses to questions that evaluated their perception of the HPV vaccine.

Six questions were posed in order to evaluate the study participant's perception of the HPV vaccine (Figure 4). When participants were asked whether a well-informed child should be able to request vaccination at sexual health clinics without parental consent, a high proportion of participants agreed but a nearly equal number of participants also disagreed. The majority of participants disagreed that HPV vaccination may encourage risky sexual behavior in adolescents. Overall, the majority of participants agreed that it was necessary to discuss issues of sexuality before recommending the HPV vaccine to adolescents and that reassurance on the efficacy and safety of the vaccine is still needed. Although more participants agreed that the HPV vaccine should be given to boys as well as girls, there was no consensus because a nearly equal number of participants disagreed and a proportion of participants were unsure.

## Discussion

A number of US studies have been conducted to address knowledge and attitudes related to HPV and the HPV vaccine. However, few are population-based. Many of these have been based on focus groups [9-11] or are studies that primarily involved a distinct racial and/or ethnic group [12,13]. A recent study of females from the US general population has been published; this study, however, did not include comparisons of knowledge and attitudes between ethnic or racial groups and geographic locations [14]. We have, for the first time, reported the assessment of HPV and HPV vaccine knowledge and perception in a sample of the general population (both males and females) in Pittsburgh, Pennsylvania and Hampton, Virginia, USA. We have compared our findings by race as well as by geographic location. Overall, our findings show that in this study population, Blacks are less knowledgeable about HPV compared with Whites. The majority of participants were unaware of important information about HPV infection, in general. Although a substantial proportion of the study population knew that HPV was sexually transmitted (78%), only 64% of Black participants were knowledgeable compared with 81% of the White participants. Furthermore, a smaller proportion of Blacks knew that both males and females could be infected with HPV (Blacks 54% vs. Whites 65%); roughly 2/3 of those interviewed knew that the incidence of HPV infection is highest among women in their 20 s and 30 s (Blacks 69% vs. Whites 65%). These data suggest that simple education might serve not only to increase awareness about the risks associated with HPV infection, but may promote changes in behavior that might lead to risk reduction of HPV infection.

Two large population-based surveys involving random samples of 1,006 participants in the US [14] and of US women, ages 18–75 years [15], reported that about 40–41% of participants knew that HPV caused cervical cancer. In our study population, the majority of participants (81%) were aware of this information, and a smaller proportion of Black participants were knowledgeable compared with Whites (73% vs. 91%). One possible explanation for the differences in reported findings between other studies and ours is that the majority of participants in our study consisted of participants with some post-secondary education. Nevertheless, since HPV is the etiologic agent for cervical and most other anogenital cancers [16-18] and has been implicated in oropharyngeal cancers [1,2], information related to HPV infection is crucial to an individual's understanding of their risk for infection and development of these serious diseases.

We compared the distribution of correct answers to the HPV knowledge questions between geographic locations and observed that overall the mean number of correct answers was higher for Hampton participants compared with Pittsburgh participants, although this difference was not statistically significant. When we compared the Black and White participants from Pittsburgh, we observed that Blacks had a smaller mean number of correct answers compared with Whites, while there was no difference between races for Hampton participants. Overall, these findings suggest a need for increased educational programs on HPV and the clinical manifestations of HPV infections for the general popualtion residing in the Pittsbugrh area as well as for Black participants living in both geographic areas.

We have reported that 87% of participants had heard of the HPV vaccine. However, despite this high proportion of participants, the majority did not know about the protective impact. Only 18% of participants knew that the HPV vaccine protected against most cervical cancer and genital warts. This suggests that the general population may not be equipped with the necessary information to make an informed decision about HPV vaccination. We also observed that Blacks were less well informed about the HPV vaccine compared with Whites. However, the differences were statistically significant only for the Pittsburgh population, suggesting a need for targeted education programs on the HPV vaccine in this group.

## Conclusion

These data suggest that although the general population might be aware of HPV and the HPV vaccine, knowledge of the benefits from the HPV vaccination may not be apparent. There were also differences in the level of knowledge based on ethnicity and geographic location. The development of educational interventions is necessary to ensure that the general public is able to make an informed decision regarding HPV vaccination.

## Competing interests

The authors declare that they have no competing interests.

## Authors' contributions

CR, ET, RPE, SMG and DEH conceived the study and participated in the study design. CR developed the survey instrument, performed the data entry and analysis. CAS and CB were responsible for overseeing the data collection at Hampton, VA. KH, EJ, CM and AS participated in the participant recruitment and data collection at Hampton, VA; JJ, AA, NET and LR participated in participant recruitment and data collection in Pittsburgh, PA. All authors read and approved the final manuscript.
